# A huge benign gastric schwannomas presented with upper and lower gastrointestinal bleeding: a case report and literature review

**DOI:** 10.1093/jscr/rjae267

**Published:** 2024-04-26

**Authors:** Mohammed N AlAli, Aya K AlDayel, Afraj T Alshammari, Mohamed S Essa, Maha AlAmodi, Muath Alrashed, Sadiq M Amer, Mohammed A Meaigel, Talal M AlTahan, Khalid S Ahmad

**Affiliations:** Department of Surgery, Prince Mohammed Bin Abdulaziz Hospital, Ministry of Health, Riyadh, Saudi Arabia; Department of Surgery, Prince Mohammed Bin Abdulaziz Hospital, Ministry of Health, Riyadh, Saudi Arabia; College of Medicine, Princess Nourah Bint Abdulrahman University, Riyadh, Saudi Arabia; Department of Surgery, Prince Mohammed Bin Abdulaziz Hospital, Ministry of Health, Riyadh, Saudi Arabia; General Surgery Department, Faculty of Medicine, Benha University, Benha, Egypt; Department of Surgery, College of Medicine, King Khalid University, Abha, Saudi Arabia; Department of Surgery, Prince Mohammed Bin Abdulaziz Hospital, Ministry of Health, Riyadh, Saudi Arabia; Department of Pathology, Prince Mohammed Bin Abdulaziz Hospital, Ministry of Health, Riyadh, Saudi Arabia; Department of Surgery, Prince Mohammed Bin Abdulaziz Hospital, Ministry of Health, Riyadh, Saudi Arabia; Department of Surgery, Prince Mohammed Bin Abdulaziz Hospital, Ministry of Health, Riyadh, Saudi Arabia; Department of Surgery, Prince Mohammed Bin Abdulaziz Hospital, Ministry of Health, Riyadh, Saudi Arabia

**Keywords:** gastric schwannoma, mesenchymal tumor, gastrointestinal bleeding, neurinoma, Schwann cells, gastric gastrointestinal stromal tumor

## Abstract

Gastric schwannomas (GS) are rare mesenchymal tumors from Schwann cells in the gastrointestinal (GI) tract, representing 2–6% of such tumors. We report a 52-year-old woman who experienced abdominal pain, hematemesis, and melena, initially suspected of having a GI stromal tumor through ultrasound and computed tomography abdomen. Despite no active bleeding found during an upper endoscopy, she underwent a successful open subtotal gastrectomy, with histopathology confirming GS. The diagnosis of GS, which may mimic other GI conditions, relies heavily on imaging and histopathological analysis due to its nonspecific symptomatology, including the potential for both upper and lower GI bleeding. This case underscores the diagnostic challenges of GS and highlights surgical resection as the preferred treatment, generally leading to a favorable prognosis.

## Introduction

Gastric schwannomas (GS) are rare gastrointestinal (GI) tumors, with around 221 cases reported globally [1]. They belong to the group of spindle cell mesenchymal tumors, which includes leiomyoma, leiomyosarcoma, and GI stromal tumor (GIST), with GISTs being the most common. GS, however, make up only 2–6% of all mesenchymal tumors [2]. They show a female-to-male ratio of 2:1 or higher, predominantly affecting individuals aged 40–60 years [3]. GSs develop slowly, often requiring surgical resection for definitive diagnosis, and mainly occur in the stomach [4]. While typically asymptomatic and benign, some cases may present with nausea, vomiting, altered bowel habits, abdominal cramps, or rarely, hematemesis or melena [5]. Malignant transformation of GS is very rare [6].

To the best of our knowledge, the simultaneous presentation of upper and lower GI bleeding secondary to GS is exceptionally rare, underscoring the global rarity of GS.

## Case report

A 52-year-old female with no significant past medical or surgical history presented to the emergency department complaining of 1-day epigastric abdominal pain. This pain was associated with one episode of vomiting, approximately 300 ml of bloody content, and one episode of melena. The pain was intermittent, not related to feeding, without radiation, and lacked relieving factors. The patient denied any previous similar episodes, history of dysphagia, bleeding from other sites, weight loss, night sweats, fever, yellowish discoloration of the sclera, skin changes, medication use, or family history of malignancy.

**Figure 1:**
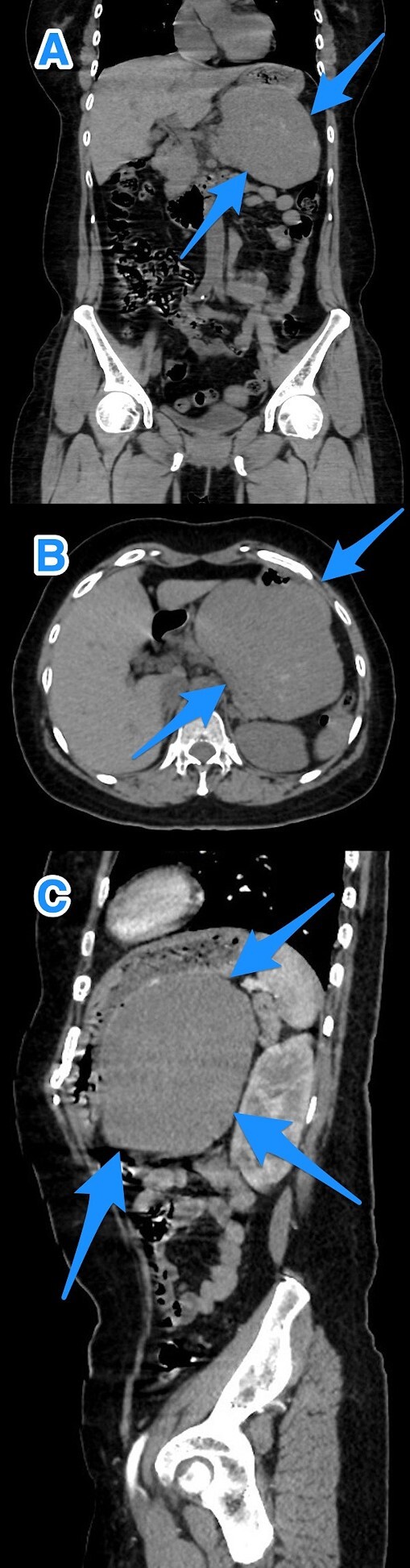
A multi-view computed tomography scan of the abdomen (A–C) showing a well-defined large gastric fundal/body submucosal heterogeneously hypoenhancing soft tissue mass lesion of the posterior gastric wall (arrows).

Upon clinical examination, the patient was alert but pale and dehydrated, with a heart rate of 125 bpm, blood pressure of 100/54 mmHg, and melena noted on rectal examination. Laboratory tests showed a leukocyte count of 13.8 x 10^9^/L, a hemoglobin level of 5.8 g/dL, and a hematocrit level of 16.8%. After resuscitation, an ultrasound showed a large hypoechoic lesion near the left liver lobe and behind the pylorus. A computed tomography (CT) scan revealed a large, well-defined mass (12.6 x 8 x 12.7 cm) in the gastric fundus/body, touching liver segment III and positioned between the stomach and pancreas, affecting the left gastric artery branches (Fig. 1).

An initial GIST diagnosis was made based on CT findings. An upper GI scope revealed a large mass at 6 o’clock, ~5 cm from the gastroesophageal junction. There was ulceration in the mass with minimal oozing, which was managed with argon plasma coagulation, epinephrine, and a gold probe.

**Figure 2:**
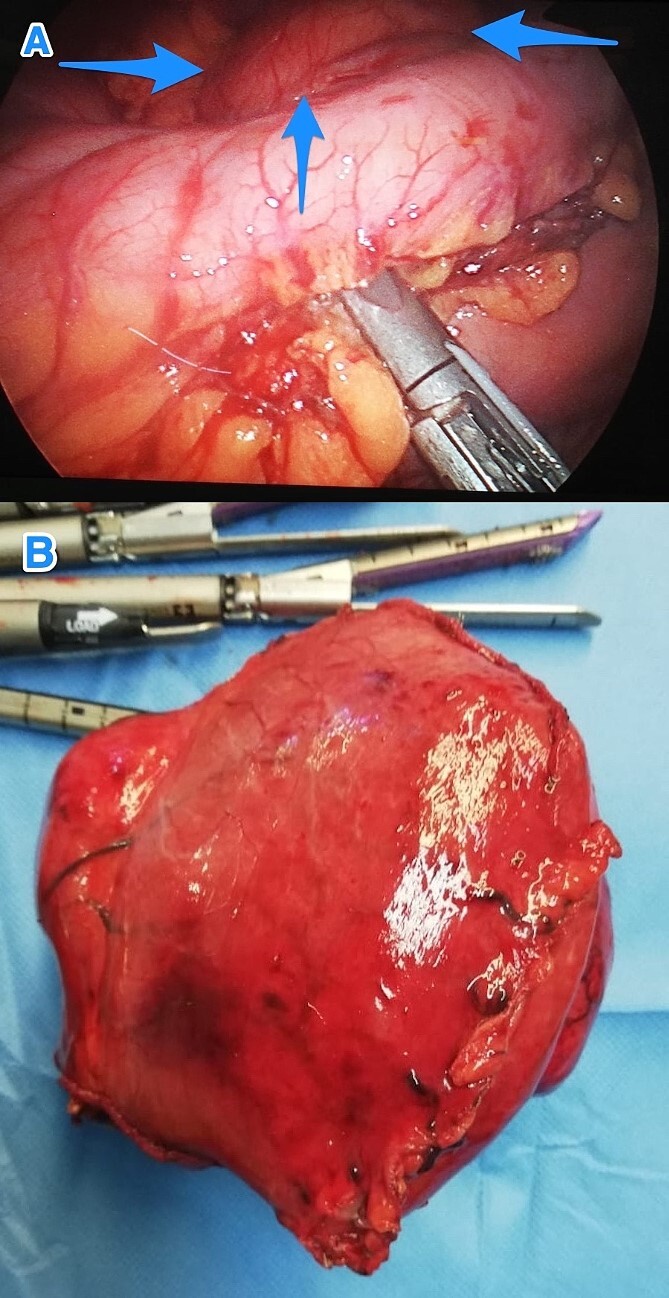
Intraoperative views of the gastric mass before (A) and after (B) resection (arrows).

**Figure 3:**
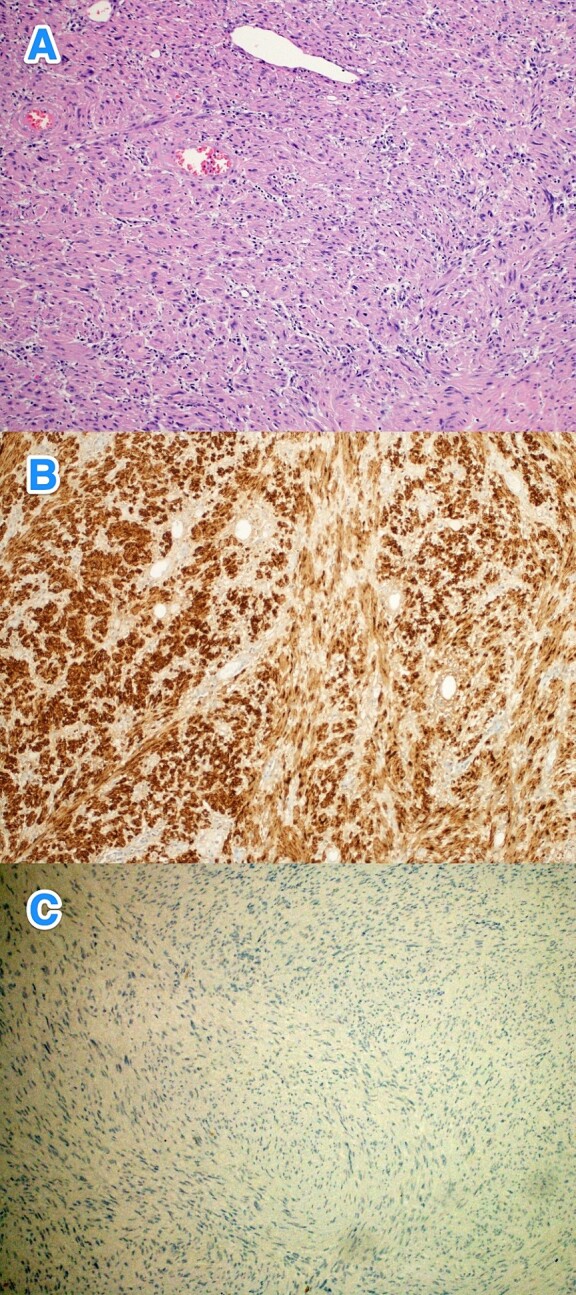
The histological sections of gastric mass show (A) a spindle cell tumor infiltrating through the gastric wall. The tumor cells are positive for S100 (B), while negative for (C) CD117, Dog-1, SMA, H-Caldesmon, Desmin, MSA, and B-Cahtenin (hematoxylin and eosin stain; 10x).

Therefore, patient underwent a laparoscopic attempt converted to an open subtotal gastrectomy with Roux-en-Y gastric bypass (RYGB) without lymph node dissection (Fig. 2). The postoperative course was uneventful, and the patient was discharged on the 12th postoperative day. Histopathological examination of the resected specimen revealed GS with a tumor size of 11 cm, negative surgical margins, and mild gastric congestion. The tumor tested positive for S100 with diffuse and strong nuclear and cytoplasmic staining and negative for B-Catenin, CD117, DOG1, SMA, H-Caldesmon, Desmin, and MSA (Fig. 3). However, the CEA level was 0.5 ng/ml. The case was discussed at the multidisciplinary tumor board, with a follow-up plan every 6 months for clinical exams and CT scans. Unfortunately, the patient was lost to follow-up 1 month after discharge.

## Discussion

GSs are uncommon, mostly benign mesenchymal tumors that grow slowly, often causing vague GI symptoms. They can be detected incidentally during endoscopy or imaging. Symptoms vary with the tumor’s size, location, and malignancy, with upper abdominal issues often due to stomach involvement [7]. Tumors growing into the mucosa can cause complications like obstruction and bleeding. Large GSs may lead to atypical symptoms such as chest pain and pleural effusion, indicating growth into nearby organs [8]. In our case, the patient's presentation with abdominal pain and upper and lower GI bleeding was likely attributable to the tumor's size and surface ulceration.

Diagnosing GS without surgery is difficult. Common serum tumor markers often remain normal, though CA19-9 may be elevated [7]. Key imaging techniques include CT scans, which show a distinct, variably enhanced mass, and endoscopic ultrasound (EUS), providing detailed information on the tumor's size, location, and features [5]. EUS-guided biopsies improve preoperative diagnosis accuracy by 10%, crucial for GI cancers. CT and upper endoscopy are important for diagnosing and planning surgery. FDG-PET is used to evaluate the tumor’s malignancy potential, recurrence, and metastasis risk, yet its preoperative diagnostic usefulness is limited [7].

Surgical resection remains the preferred treatment for GS, with laparoscopic or open approaches selected based on the tumor's size and location [1, 7]. Routine lymph node dissection is generally not recommended due to the infrequent occurrence of lymph node metastases. For tumors smaller than 3 cm, endoscopic excision is advisable, albeit with increased risks of bleeding, perforation, and GI fistulas due to submucosal tumor growth. There is no conclusive evidence that endoscopic resection leads to incomplete tumor removal, recurrence, or metastasis [7]. In this case, complete tumor resection was achieved through open approach. Histopathologically, GS is identified by spindle-shaped cells with elongated nuclei and a wavy cytoplasmic pattern, staining positively for S-100 protein and vimentin, while typically negative for CD117, DOG1, SMA, and H-Caldesmon [6]. This case's tumor exhibited positive staining for S-100 protein and negativity for CD117, DOG1, SMA, and H-Caldesmon, confirming a GS diagnosis.

The prognosis for GS is generally favorable, with a low recurrence and metastasis risk [7, 8]. However, factors such as larger tumor size and proximal stomach location may increase the likelihood of malignant transformation. Malignant schwannomas carry a poor prognosis, with variable chemotherapy responses and a 5-year survival rate of only 23%. Long-term follow-up, including clinical exams and CT scans every 6 months, is recommended to monitor for recurrence or malignant transformation [9]. Unfortunately, in this case, the patient was lost to follow-up 1-month post-discharge.

## Conclusions

GS, a rare tumor, can present with vague symptoms like GI bleeding or be found incidentally on imaging. Accurate diagnosis requires careful histopathological examination. Surgical resection is the treatment of choice, often leading to a good prognosis.

## Conflict of interest statement

None declared.

## Funding

None declared.

## Patient consent

Informed consent for publication was taken from the patient.
